# Annual acknowledgement of manuscript reviewers

**DOI:** 10.1186/1472-6920-13-40

**Published:** 2013-04-05

**Authors:** Adrian Aldcroft

**Affiliations:** 1 BioMed Central, Floor 6, 236 Gray's Inn Road, London, WC1X 8HB, UK

## Abstract

**Contributing reviewers:**

The editors of *BMC Medical Education* would like to thank all our reviewers who have contributed to the journal in Volume 12 (2012).

## 

Eva Aagaard

USA

Hamza Abdulghani

Saudi Arabia

Adrienne Ables

USA

Marwan Abu-Hijleh

Bahrain

Francis Achike

USA

Dimitri Aerden

Belgium

Adriana Aguiar

Brazil

Rola Ajjawi

United Kingdom

Hanan Al Kadri

Saudi Arabia

Jawad Al-Diwan

Iraq

Ana Allen Gomes

Portugal

Abtin Alvand

United Kingdom

Nkeiruka Ameh

Nigeria

Seyi Ladele Amosun

South Africa

Mark Archambault

USA

Julian Archer

United Kingdom

Douglas Archibald

Canada

Neil Arya

Canada

Martine Baars

Netherlands

Christine Baatz

Germany

Marilyn Baird

Australia

Keith Baldwin

USA

Thomas Balslev

Denmark

Daniel Banks

USA

Amanda Barnard

Australia

Peter Baume

Australia

Jochanan Benbassat

Israel

Deirdre Bennett

Ireland

Norman Berman

USA

Ian Bickle

United Kingdom

Beth Bierer

USA

Yvonne Birks

United Kingdom

Alan Bleakley

United Kingdom

Phil Blyth

New Zealand

Beatrice Boateng

USA

Valdes Bollela

Brazil

Adrian Boswood

United Kingdom

Jean-Pierre Bourguignon

Belgium

Judith Bowen

USA

Anja M Braend

Norway

Chantal Brazeau

USA

Jennifer Breckler

USA

Adrian Brooke

United Kingdom

Ryan Brydges

Canada

Sharon Buckley

United Kingdom

Simon Budd

United Kingdom

Vanessa Burch

South Africa

Andrew Butler

USA

Jeff Cain

United States Minor Outlying Islands

Kay Caldwell

United Kingdom

Ben Canny

Australia

Ted Carnevale

USA

Sandra Carr

Australia

Paulo Carvalho-Junior

Brazil

Afonso Cavaco

Portugal

Nora Celebi

Germany

Elijah Chaila

Ireland

John Chambers

United Kingdom

Bernard Charlin

Canada

Carolyn Charman

United Kingdom

Belinda Chen

USA

Shu-Wen Chen

Taiwan

Walter Chen

Taiwan

Kun-Yang Chuang

Taiwan

Mike Clapham

United Kingdom

Jennifer Cleland

United Kingdom

Stuart Cohen

United Kingdom

Janke Cohen-Schotanus

Netherlands

Fabrizio Consorti

Italy

Deborah Corner

United Kingdom

Selma Corovic

Slovenia

Manuel Costa

Portugal

Laura Cotlin

USA

Desiree Cox-Maksimov

United Kingdom

Gerda Croiset

Netherlands

Pat Croskerry

Canada

Vinette Cross

United Kingdom

Jim Crossley

United Kingdom

Sheila Crow

USA

Giuseppe Curcio

Italy

Francois Dabis

France

Ruth Dalemans

Belgium

Ivan Darby

Australia

Ben Darlow

New Zealand

Prithwiraj Das

United Kingdom

W. Dale Dauphinee

Canada

Subodh Dave

United Kingdom

Brian Davies

United Kingdom

Ashlesha Dayal

USA

Patrick De Maziere

Belgium

Wouter De Ruijter

Netherlands

Martjie De Villiers

South Africa

Hugo De Waal

United Kingdom

Linda Decherrie

USA

Clare Delany

Australia

George Delclos

USA

Reg Dennick

United Kingdom

Petra Diaz Del Campo

Spain

Ioannis Dimoliatis

Greece

Simone Diniz

Brazil

Agnes Dodds

Australia

Eva Doherty

Ireland

D Dolmans

Netherlands

Eleanor Dommett

United Kingdom

Jon Dowell

United Kingdom

Erik Driessen

Netherlands

Angela Durey

Australia

Samuel Edelbring

Sweden

Gudrun Edgren

Sweden

Berit Eika

Denmark

N Eizenberg

Australia

Charles Eldridge

USA

Carrie Elzie

USA

Katarzyna Emerich

Poland

Carlos Estrada

USA

Liz Farmer

Australia

Ramon Fayad

Colombia

Tim Fedak

Canada

Christopher Feddock

USA

James Feldman

USA

Lynette Fernandes

Australia

Luis Fernandez Luque

Norway

Cayetano Fernández-Sola

Spain

Max Field

United Kingdom

Simon Field

Canada

Richard Fiordo

USA

Marina Fiori

Switzerland

Sabine Fischbeck

Germany

Lisa Fleet

Canada

Amy Fleming

USA

Ian Fletcher

United Kingdom

Uno Fors

Sweden

Brenda Fraser

Canada

Kristin Fraser

Canada

Tracy Frech

USA

Keith Frey

USA

Peter Gallagher

Nigeria

Lisa Genzel

United Kingdom

Waltraud Georg

Germany

Joseph Geskey

USA

Sarmishtha Ghosh

Malaysia

Anne Gill

USA

Elisabeth Gjerberg

Norway

Helen Goodyear

United Kingdom

Marjan Govaerts

Netherlands

Aysegul Gozu

USA

Andrew Grant

United Kingdom

Janet Grant

United Kingdom

Ian Green

Australia

Margarete Grimus

Austria

Gregory Gruener

USA

Tore Gude

Norway

Cally Guerin

Australia

Erol Gurpinar

Turkey

Anthony Hagger

United Kingdom

Hossam Hamdy

United Arab Emirates

Wolfgang Hampe

Germany

Janice Hanson

USA

Inam Haq

United Kingdom

Sigrid Harendza

Germany

Spencer Harpe

USA

Jeff Harrison

New Zealand

Neil Harrison

United Kingdom

Jo Hart

United Kingdom

Rose Hatala

Canada

John Hattie

Australia

Leonhard Held

Switzerland

Marcus Henning

New Zealand

Carol Herbert

Canada

Rahmatina Herman

Indonesia

Andrew Hill

New Zealand

Nancy Hills

USA

George Hogg

United Kingdom

Tanis Hogg

USA

Andreas Holzinger

Austria

Andreas Holzinger

Austria

Mary Howman

United Kingdom

Susan Hrisos

United Kingdom

Judith N (Nicky) Hudson

Australia

Jeffrey Huebner

USA

Thomas Hughes

United Kingdom

Tristan Huie

USA

Adrian Husbands

United Kingdom

Sören Huwendiek

Germany

Robert Ingram

United Kingdom

Debra Jackson

Australia

Henry James

Bahrain

Aminah Jatoi

USA

Mariëlle Jippes

Netherlands

Jakob Johansson

Sweden

Peter Johnston

United Kingdom

Anne Jones

Australia

Nathan Jones

USA

Nicole Jones De Rooy

Australia

Anne Marie Kanstrup

Denmark

Orit Karnieli-Miller

Israel

Aliya Kassam

Canada

William Keenan

USA

Clare Kell

United Kingdom

Vikas Khanduja

United Kingdom

Priya Khanna

Australia

Ee Ming Khoo

Malaysia

Claudia Kiessling

Switzerland

Sue Kilminster

United Kingdom

Michael Kim

USA

Gail Kinman

United Kingdom

Alexander Kiss

Switzerland

Mary Kite

USA

Matthias Knobe

Germany

Joseph Kolars

USA

Anneke Kramer

Netherlands

Kristian Krogh

Denmark

Frederick Kron

USA

Edward Krupat

USA

Jan Kuks

Netherlands

Igor Lackovic

Croatia

Fred Lado

USA

Nai Ming Lai

Malaysia

Madhab Lamsal

Nepal

Christopher Landrigan

USA

James Lange

USA

Wolf Axel Langewitz

Switzerland

Young-Mee Lee

Korea South

Sam Leinster

United Kingdom

Jeff Leiter

Canada

Zorayda Leopando

Philippines

Felix Leung

USA

Leah Levi

USA

David Lewis

United Kingdom

Jinping Li

USA

Jie Liang

China

Victor Lim

Malaysia

Dileep Lobo

United Kingdom

Jocelyn Lockyer

Canada

Ruud Loffeld

Netherlands

Andrew Long

United Kingdom

Marios Loukas

Grenada

Heather Loveday

United Kingdom

Stuart Lubarsky

Canada

Katarzyna Ludka

United Kingdom

Andrew Luxton-Reilly

New Zealand

Irene Ma

Canada

Herve Maisonneuve

France

Gerardus Daniël Majoor

Netherlands

Moira Maley

Australia

Bente Malling

Denmark

Seth Manoach

USA

Donald Marcus

USA

Stuart Marshall

Australia

Stephen Mason

United Kingdom

Maria Mathews

Canada

Marie Matte

Canada

Brian Mavis

USA

Win May

USA

Patricia Mcburney

USA

Louise Mccall

Australia

Elizabeth Mcclure

USA

Sindy Mccrystle

USA

Patricia Mcinerney

South Africa

Scott Mcintosh

USA

Danette Mckinley

USA

Chris Mcmanus

United Kingdom

John Mcnulty

USA

Ritesh G Menezes

India

Alan Merry

New Zealand

Jamie Meuser

Canada

Larry Michaelsen

USA

Susan Miles

United Kingdom

Gary Misan

Australia

Theresa Mitchell

United Kingdom

Andile Mji

South Africa

Susan Moch

USA

Willemina Molenaar

Netherlands

Alireza Monajemi

Iran

Carlton Moore

USA

Norman Morris

Australia

Todd Morrison

Canada

Elizabeth Morrow

United Kingdom

Christine Moutier

USA

Deborah Murdoch-Eaton

United Kingdom

Bruce Newton

USA

Honor Nicholl

Ireland

Katri Nieminen

Sweden

Christoph Nikendei

Germany

Jonas Nordquist

Sweden

Robert Norman

United Kingdom

Pauline Norris

New Zealand

Romer Ocanto

USA

Zeliha Ocek

Turkey

Elke Birgit Ochsmann

Germany

Erkki Olkinuora

Finland

Hirotaka Onishi

Japan

Michelle O'Reilly

United Kingdom

Elena Oris (Kourdioukova)

Belgium

Jay Orlander

USA

Jay Orlander

USA

Robin Osborn

USA

Christoph Ostgathe

Germany

Helen O'Sullivan

United Kingdom

Patricia O'Sullivan

USA

Alis Ozcakir

Turkey

Gordon Page

Canada

Edward Palmer

Australia

Alexia Papageorgiou

United Kingdom

Evridiki Papastavrou

Cyprus

Tracey Papinczak

Australia

Helena Paro

Brazil

Christina Passali

Greece

Carl Patow

USA

Adam Paul

United Kingdom

John Peabody

USA

Godfrey Pell

United Kingdom

Helen Perry

USA

Ray Peterson

Australia

Huy Phan

Australia

Ralph Pinnock

Australia

Gominda Ponnamperuma

Sri Lanka

Phillippa Poole

New Zealand

Rebekah Pratt

USA

Helena Priest

United Kingdom

Carla Pugh

USA

Thelma Quince

United Kingdom

Gandes Retno Rahayu

Indonesia

Gopinath Ranjith

United Kingdom

Daphne Rattner

Brazil

Saleem Razack

Canada

Tom Reader

United Kingdom

Jennifer Reath

Australia

Harold Reiter

Canada

Jean-Marie Renard

France

Nathaniel Rickles

USA

Simon Riley

United Kingdom

Elianne Riska

Finland

Farwa Rizwi

Pakistan

William Roberts

USA

Stephen Robson

Australia

David Rodgers

USA

Charles Christoph Roehr

Germany

David Rogers

USA

Gary Rogers

Australia

Bruna Maria Rondinone

Italy

Marcy Rosenbaum

USA

Linette Ross

USA

Arie Rotem

Australia

Jonathan Round

United Kingdom

Colin Royse

Australia

Joy Rudland

New Zealand

Miriam Ruesseler

Germany

Kathy Rush

Canada

Cristin Ryan

Ireland

Charlotte Salter

United Kingdom

Paul Sambrook

Australia

Wallee Satayasai

Thailand

Jason Satterfield

USA

Anurag Saxena

Canada

Albert Scherpbier

Netherlands

Angelika Schlarb

Germany

Christopher Schlett

Germany

Michal Schneider-Kolsky

Australia

Georgina Selby

United Kingdom

Tarun Sen Gupta

Australia

Yesim Senol

Turkey

Reginald P. Sequeira

Bahrain

Patricia Sexton

USA

Naiemeh Seyedfatemi

Iran

P Ravi Shankar

Nepal

Saran Shantikumar

United Kingdom

Ben Shaw

United Kingdom

Dale Sheehan

New Zealand

Marwan Sheikh-Taha

Lebanon

Angela Shepherd

USA

Eun-Jung Shim

Korea South

Boaz Shulruf

Australia

Anne Simmenroth-Nayda

Germany

Arjun Singh

India

Nalini Singhal

Canada

Royce Singleton

USA

Bonnie Smith

USA

Claire Smith

United Kingdom

Helen Smith

United Kingdom

David Snadden

Canada

David Solomon

USA

George Somers

Australia

Marlee Spafford

Canada

Cord Spreckelsen

Germany

Sara Stafford

Canada

Anke Steckelberg

Germany

Anne Stephenson

United Kingdom

Deborah Stiffler

USA

Hugh Stoddard

USA

Kathrin Stoll

Canada

Peter Stone

New Zealand

Roger Strasser

Canada

Angela Taft

Australia

Masami Tagawa

Japan

Kasra Taherian

United Kingdom

Gordon Tait

Canada

Ann Taket

Australia

Mohsen Tavakol

United Kingdom

Beck Taylor

United Kingdom

Celia Taylor

United Kingdom

David Taylor

United Kingdom

Holly Taylor

USA

Amy Teten

USA

Julie K. Tilson

USA

Anne Tonkin

Australia

David Topps

Canada

Richard Tubman

United Kingdom

Ingrid Tyler

Canada

Linda Vallino

USA

Renée Van Der Leeuw

Netherlands

Jouke Van Der Zee

Netherlands

Nynke Van Dijk

Netherlands

Tamara Van Gog

Netherlands

Marc Van Nuland

Belgium

Jan Van Tartwijk

Netherlands

Chris Van Weel

Netherlands

Judith Venuti

USA

Pirashanthie Vivekananda-Schmidt

United Kingdom

Kevin Volkan

USA

Birgitta Wallstedt

Denmark

Allyn Walsh

Canada

Jason Ward

United Kingdom

Julia Ward

USA

Anthony Warrens

United Kingdom

Simon Watmough

United Kingdom

Alan Weindling

United Kingdom

Jennifer Mary Weller

New Zealand

Gig Wernbacher-Searle

Austria

Anne Werner

Germany

Maria Weurlander

Sweden

Peter Weyrich

Germany

Jonathan White

Canada

Sue Whittle

United Kingdom

Doni Widyandana

Indonesia

John Wiecha

USA

Lionel Wijesuriya

Malaysia

Lisette Wijnia

Netherlands

Ann Wilkinson

United Kingdom

David Wilkinson

Australia

Tim Wilkinson

New Zealand

Brent Williams

USA

Jane Williams

United Kingdom

John Williams

Canada

Marie Williams

Australia

Ian Wilson

Australia

Jeffrey Wiseman

Canada

Fredric Wolf

USA

Roger Wong

Canada

Katherine Woolf

United Kingdom

Fe-Lin Lin Wu

Taiwan

Janet Yates

United Kingdom

Terri Yates

USA

Jianzhen Zhang

Australia

Tadeusz Zielonka

Poland

Craig Zimitat

Australia

## Pre-publication history

The pre-publication history for this paper can be accessed here:

http://www.biomedcentral.com/1472-6920/13/40/prepub

